# Effects of Glioblastoma Resection on Cognitive Function and Affective Symptoms at Three‐Month Follow‐Up

**DOI:** 10.1002/brb3.71478

**Published:** 2026-05-05

**Authors:** Carina E. Tschirky, David Bellut, Nicolin Hainc, Ramona Hauser, Anne‐Katrin Hickmann, Andre Richter

**Affiliations:** ^1^ Department of Consultation‐Liaison‐Psychiatry and Psychosomatic Medicine University Hospital Zurich, University of Zurich Zurich Switzerland; ^2^ Department of Internal Medicine Stadtspital Waid Zurich Switzerland; ^3^ Department of Neurosurgery, Clinical Neuroscience Center University Hospital Zurich Zurich Switzerland; ^4^ Department of Neuroradiology, Clinical Neuroscience Center University Hospital Zurich, University of Zurich Zurich Switzerland; ^5^ Department of Neurology, Inselspital Bern University Hospital, University of Bern Bern Switzerland; ^6^ Neurochirurgisches Zentrum Ostschweiz St. Gallen Switzerland; ^7^ Department of Neurosurgery Kantonsspital St. Gallen St. Gallen Switzerland

**Keywords:** affective symptoms, anxiety, cognitive function, depression, glioblastoma, lateralization, location, resection

## Abstract

**Background:**

Glioblastoma (GBM) is the most frequent primary malignant brain tumor characterized by aggressive growth and poor prognosis, frequently accompanied by cognitive and affective deficits that severely impair quality of life (QoL). However, the short‐term course of these cognitive and emotional functions after surgical resection remains insufficiently understood.

**Aims:**

This study aimed to investigate changes in cognitive performance and affective symptoms before and 3 months after GBM resection and to explore the influence of tumor characteristics such as lateralization and location. We hypothesized that cognitive function and affective symptoms would improve or remain stable postoperatively and that tumor‐related factors would modulate these trajectories.

**Methods:**

In this multicenter pre–post observational study, 37 adults with histopathologically confirmed GBM (World Health Organization [WHO] 2021) were assessed using the Montreal Cognitive Assessment (MoCA) and the Hospital Anxiety and Depression Scale (HADS). Paired *t*‐tests, Wilcoxon tests, ANOVA, and linear regression were conducted to evaluate pre‐ to postoperative changes and correlations with tumor characteristics.

**Results:**

Cognitive performance remained stable after resection. Although the HADS total score showed no significant pre‐ to postoperative change, patients with preoperative anxiety or depressive symptoms demonstrated significant postoperative improvement (HADS‐A *p =* 0.010; HADS‐D *p =* 0.012). Higher preoperative HADS scores predicted greater symptom reduction. Higher affective burden was shown in patients with right‐hemispheric and parieto‐occipital tumors, while temporal lobe tumors were associated with anxiety that decreased after resection (*p =* 0.009).

**Conclusions:**

GBM resection maintained cognitive function while improving affective symptoms in patients with elevated preoperative distress. Systematic psychological screening and tailored psychosocial interventions may enhance emotional resilience and QoL in GBM care.

## 1 Background

Glioblastoma (GBM) is the most frequent primary malignant brain tumor in adults, with an incidence of 3.23 per 100,000 population. It represents the most aggressive form of primary brain neoplasms, characterized by highly infiltrative growth, rapid progression, and poor prognosis despite multimodal therapeutic approaches (Ostrom et al. 2020; Stupp et al. 2009). The course of the disease includes not only the devastating neurological and medical consequences of tumor infiltration, but also significant psychosocial and cognitive challenges. Hence, GBM results in a significant reduction in patients’ quality of life (QoL) (Solanki et al. 2017; Alexander and Cloughesy 2017).

Cognitive deficits are highly prevalent in GBM patients (Tucha et al. 2000; Rijnen et al. 2020) and have been shown to substantially compromise daily functioning and overall QoL (Solanki et al. 2017), thereby contributing significantly to the burden of disease. Various causes of cognitive decline have been described, including direct tumor effects, peritumoral edema, increased intracranial pressure, and treatment‐related factors (Dallabona et al. 2017; Taphoorn and Klein 2004).

In addition to cognitive deficits, affective symptoms are also frequent in this population (Mugge et al. 2020). Anxiety and depression can impair daily functioning, creating a significant burden for both patients and their caregivers (Rooney et al. 2011; Au et al. 2022). Furthermore, it has been demonstrated that they are an important independent predictor of QoL (Pelletier et al. 2002) and overall survival, with a clinically important correlation between affective symptoms and GBM prognosis (Fu et al. 2020).

The relationship between tumor characteristics, cognitive performance, and the development of affective symptoms in GBM patients is still not fully understood. Existing evidence suggests that the severity of symptoms may be influenced by preoperative conditions, tumor lateralization, and tumor location (Mainio et al. 2003; Goebel et al. 2013). Although several studies have investigated the relationship between tumor characteristics, cognitive functions, and affective symptoms, longitudinal analyses conducted in strictly homogeneous GBM cohorts remain scarce. In particular, research that systematically examines pre‐ to postoperative changes across both cognitive and affective domains within the same patients is still limited. Nevertheless, understanding these relationships is of great clinical importance, as it can help identify patients at risk, improve prognosis assessment, and develop targeted interventions. Furthermore, integrative studies linking cognitive and affective symptoms may further support the development of more effective treatment strategies.

The aim of this study is to address these gaps by systematically examining the effects of GBM characteristics, cognitive performance, and affective symptoms both preoperatively and postoperatively.

We hypothesize that: (1) cognitive performance and affective symptoms improve or stabilize following resection; and (2) cognitive and affective domains are interconnected, with their course influenced by tumor‐related factors such as lateralization and location.

By clarifying these relationships, we aim to provide information for the development of personalized interventions that address both cognitive and affective domains over the course of treatment and ultimately improve patients' psychological well‐being and QoL.

## 2 Methods

### 2.1 Study Design and Participants

In this single‐group pre–post observational multicenter study, we investigated patients with suspected GBM both immediately before and 3 months after tumor resection. Between July 2018 and February 2023, adult patients admitted to the Department of Neurosurgery at the University Hospital of Zurich and the Cantonal Hospital of St. Gallen for surgical management of a suspected high‐grade glioma were screened for eligibility to participate in the study. The inclusion criteria were (1) suspected cerebral glioma World Health Organization (WHO) grade 4 before resection; (2) age ≥ 18 years; (3) fluent in German; and (4) condition of the patient and location of the tumor were acceptable for resection. The further exclusion criteria were (1) pregnant or lactating female patients; (2) patients with chronic neurological or neuropsychiatric comorbidities; and (3) patients with current substance or alcohol abuse. Informed consent was obtained from all participants before participation, and the study was approved by the Ethics Committee of Zurich (project number 2018‐00024).

### 2.2 Sociodemographic and Clinical Characteristics

**FIGURE 1:**
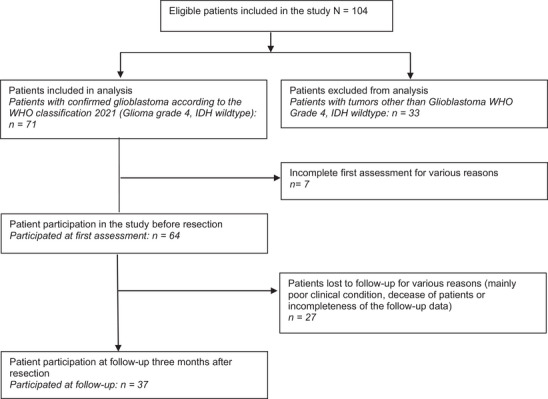
Patient flow through the study. Patients were included if they had histologically confirmed glioblastoma and completed pre‐ and postoperative assessments.

Sociodemographic data were collected from the admission reports. Suspicion of glioma was established after contrast‐enhanced MRI examination. Evaluation of the tumor lateralization and specific anatomical location was conducted by a consultant of the Department of Neuroradiology at the University Hospital of Zurich. After resection, the diagnosis was histopathologically confirmed. Patients with any diagnosis other than GBM based on the 2021 WHO classification (inclusion: diffuse glioma grade 4, IDH wild‐type; patients enrolled prior to the 2021 revision were reclassified accordingly) were excluded from the study at this point (excluded *n* = 33). All patients received standardized adjuvant treatment according to the Stupp protocol, consisting of concomitant temozolomide and radiotherapy followed by adjuvant temozolomide (Stupp et al. 2009). Figure 1 presents further details about the study's inclusion and exclusion process.

### 2.3 Cognitive Assessment

The cognitive status was assessed after admission of the patient to the hospital and 3 months after resection (follow‐up) using the Montreal Cognitive Assessment (MoCA). This is a well‐established and validated brief cognitive screening tool considered to be highly sensitive and specific for early detection of mild cognitive impairment (Nasreddine et al. 2005). The test can be divided into six subscales assessing six different cognitive domains of the brain. The areas tested include (1) visuospatial abilities using a clock‐drawing task and a three‐dimensional cube copy; (2) executive functions involving an adapted Trail Making B task, a two‐item verbal abstraction task and a phonemic fluency task and; (3) language being evaluated by using an item confrontation naming task with three different low‐familiarity animals, repetition of two syntactically complex sentences and again the phonemic fluency task; (4) attention including a continuous tapping‐test, a serial descending subtraction task and repetition of five and three digits forward and backward respectively; (5) short‐term memory involving memorizing five nouns within two trials and recalling them after approximately 5 min; and (6) the orientation to time and place being assessed at the end of the test. A score below 24 is generally considered indicative of cognitive impairment, with a minimal clinically important difference established at 2 points for meaningful clinical interpretation (Malek‐Ahmadi and Nikkhahmanesh 2024; Wong et al. 2017).

### 2.4 Anxiety and Depression Assessment

The Hospital Anxiety and Depression Scale (HADS) was used to assess affective symptoms. This questionnaire was developed to identify states of anxiety and depression in patients in nonpsychiatric departments. A total of 14 items are displayed in the HADS, whereby seven items are used for anxiety screening (HADS‐A), and another seven items are used for depression assessment (HADS‐D). Each item was scored from 0 to 3 points, resulting in a subscore range of 0 to 21 points. Referring to Bjelland et al. (2002), a HADS‐A score ≥ 8 points was defined as an anxious state and a HADS‐D score ≥ 8 points as a depressive state, respectively. Every participant completed the HADS upon admission and 3 months after resection during follow‐up. The 3‐month follow‐up time point was chosen to allow for neurological stabilization after resection and to assess patients during early adjuvant treatment. Immediate postoperative assessment was not performed, as cognitive and affective measures in the early postoperative phase may be influenced by transient factors such as cerebral edema, fatigue, medication effects (e.g., steroids, analgesics), and risk of delirium.

### 2.5 Statistical Analysis

All analyses were performed using IBM SPSS statistical software (SPSS version 29; SPSS, Chicago, IL). First, descriptive statistics were used to determine all variables depending on their level of measurement. Categorical data were expressed as absolute and relative frequencies (percentages). Continuous data were described using arithmetic means, standard deviation, and either minimum‐to‐maximum range or the median. Normality of continuous variables was assessed using visual inspection of histograms and Q–Q plots as well as the Shapiro–Wilk test. Where assumptions of normality were met, parametric tests were applied. Where normality was violated, nonparametric alternatives were used. For paired‐sample *t*‐tests, normality was assessed on the difference scores.

To compare the means of the MoCA total and subscores. representing the six cognitive domains, right before and 3 months after resection, a Wilcoxon signed‐rank test was conducted. Furthermore, we analyzed the change in HADS total and subscale scores from preoperative assessment to 3 months post‐resection using a paired‐sample *t*‐test. This analysis was conducted for the entire study population as well as for subgroups, in which participants were classified as depressive vs. nondepressive and anxious vs. nonanxious, respectively, based on their HADS score. The same test was employed to assess the impact of tumor location on HADS scores both preoperatively and postoperatively.

A linear regression analysis was conducted to assess the adjusted effect of the preoperatively attained score in the HADS assessment on the change in score pre‐ vs. postoperatively. Therefore, we divided the HADS score into the total score and the subscores for depression HADS‐D, and anxiety HADS‐A.

Furthermore, to show the comparison and effect of the tumor lateralization on the means of the preoperative HADS total and subscores, one‐way ANOVA test was conducted.

The primary outcomes of the study were defined a priori as the pre‐ to postoperative changes in MoCA total score and HADS total score. Subdomain and subgroup analyses were predefined as exploratory. Given the exploratory nature of these analyses and the small sample size, no formal correction for multiple comparisons (e.g., Bonferroni) was applied, as this could mask clinically meaningful effects. The results of the subgroup analyses should therefore be interpreted with caution, emphasizing effect direction and clinical plausibility rather than relying solely on *p* values.

## 3 Results

### 3.1 Sample Characteristics

A total of 37 participants were included in the study, all of whom had received the same histopathological diagnosis of GBM, ensuring a homogeneous study population. The characteristics of the participants are displayed in Table 1. The gender distribution was unequal, with three‐quarters of participants being male, which corresponds to the 1.6 times higher incidence of GBM in males compared to females (Carrano et al. 2021). Tumor lateralization was evenly distributed, with most tumors located in the temporal lobe (46%) and in the frontal lobe (22%).

**TABLE 1:** Sociodemographic and clinical characteristics of the study sample (*n* = 37).

Age (years)	
Mean ± SD	65.05 ± 9.40
Range	48–86
Sex (*n* [%])	
Female	9 (24.3)
Male	28 (75.7)
Tumor location (*n* [%])	
Frontal lobe	8 (21.6)
Limbic system	1 (2.7)
Temporal lobe	17 (45.9)
Parieto‐occipital lobe	5 (13.5)
Multiple locations	6 (14.3)
Tumor lateralization (*n* [%])	
Right	17 (45.9)
Left	19 (51.4)
Not recorded	1 (2.7)

*Note*: Data are presented as mean ± SD or *n* (%).

Abbreviation: SD, standard deviation.

### 3.2 Cognitive Performance and Functional Status

The analysis showed no significant changes in the MoCA total score (preoperative mean ± SD: 22.95 ± 5.5 vs. postoperative: 23.11 ± 5.6; *p =* 0.725) or in any of its subscales 3 months after GBM resection. Across all seven domains, the mean difference between pre‐ and postoperative scores was minimal (≤ 0.3 points), and the median scores remained identical at both time points. Executive functions (2.78 ± 1.1 vs. 2.76 ± 1.1), visuospatial abilities (3.08 ± 1.2 vs. 3.14 ± 1.3), attention (4.86 ± 1.6 vs. 5.05 ± 1.3), and memory (2.42 ± 1.9 vs. 2.59 ± 2.0) showed small, nonsignificant postoperative increases (all *p* > 0.52), while language scores decreased slightly (4.64 ± 1.4 vs. 4.38 ± 1.4; *p =* 0.296). Orientation remained stable (5.58 ± 0.9 vs. 5.59 ± 0.9; *p =* 0.861). The variability of the results was comparable across all time points, with nearly identical standard deviations throughout. Linear regression analysis revealed that age was significantly associated with preoperative MoCA total score (*β* = −0.229, *p =* 0.016), indicating that older patients tended to present with lower baseline cognitive performance. However, age showed no significant association with postoperative MoCA scores or with the change in MoCA scores from pre‐ to postoperative assessment, suggesting that age did not influence cognitive trajectory following resection (Supporting Information 1). Consistent with the cognitive findings, functional status assessed with the Karnofsky Performance Scale (KPS) showed no significant change from pre‐ (mean ± SD: 2.59 ± 1.19) to postoperative assessment (2.25 ± 0.88; *p =* 0.227). Overall, both cognitive performance and functional status remained stable after tumor resection.

### 3.3 Affective Symptoms

#### 3.3.1 HADS Total Score

No significant differences were found in HADS total score before and after resection for the overall cohort with the preoperative mean ± SD: 10.76 ± 7.0 vs. postoperative: 9.43 ± 6.67; *p =* 0.207. Table 2 demonstrates that when analyzing subgroups, nondepressive participants (HADS‐D < 8) as well as nonanxious participants (HADS‐A < 8) showed no significant postoperative changes in HADS total scores. In contrast, participants with depressive symptoms (HADS‐D ≥ 8) showed a significant postoperative reduction (*p =* 0.048), as did those with anxiety symptoms (HADS‐A ≥ 8; *p =* 0.025). These findings suggest that the overall trend of reduction in HADS total scores, although not significant, is primarily driven by changes in subgroups with elevated preoperative symptom levels.

**TABLE 2:** Change in score for the subgroups in HADS total scores and subscores.

	Preoperative score	Postoperative score	Change in score (pre‐ vs. postoperative)		
	Mean ± SD	Mean ± SD	*T*	95% CI	*p* (two‐sided)
Nondepressive patients (*n* = 30)					
HADS total score	8.13 ± 4.46	8.00 ± 5.98	0.130	−1.96 – 2.22	0.897
HADS‐D	3.00 ± 2.13	4.03 ± 3.14	−1.924	−2.13 – 0.07	0.064
HADS‐A	5.13 ± 3.06	3.97 ± 3.34	1.725	−0.22 – 2.55	0.095
Depressive patients (*n* = 7)					
HADS total score	22.00 ± 3.65	15.57 ± 6.32	2.482	0.09 – 12.77	0.048*
HADS‐D	11.29 ± 2.90	7.71 ± 3.68	3.584	1.13 – 6.01	0.012*
HADS‐A	10.71 ± 2.81	7.86 ± 3.08	1.713	−1.22 – 6.94	0.138
Nonanxious patients (*n* = 26)					
HADS total score	7.38 ± 4.23	7.62 ± 6.46	−0.207	−2.53 – 2.07	0.838
HADS‐D	3.08 ± 2.80	4.04 ± 3.55	−1.593	−2.21 – 0.28	0.124
HADS‐A	4.31 ± 2.36	3.58 ± 3.31	0.978	−0.81 – 2.27	0.338
Anxious patients (*n* = 11)					
HADS total score	18.73 ± 5.52	13.73 ± 5.22	2.642	0.78 – 9.22	0.025*
HADS‐D	8.09 ± 4.04	6.36 ± 2.94	1.652	−0.60 – 4.06	0.129
HADS‐A	10.64 ± 2.16	7.36 ± 2.84	3.157	0.96 – 5.58	0.010**

*Note*: Data are presented as mean ± SD.

Abbreviations: CI, confidence interval, HADS, hospital anxiety and depression scale; HADS‐D, depression subscale; HADS‐A, anxiety subscale; *T*, *t*‐statistic.

*p ≤ 0.05.

**p ≤ 0.01.

#### 3.3.2 HADS‐A

HADS‐A scores were significantly lower 3 months postoperatively (mean ± SD 4.7 ± 3.6) compared to preoperative scores (mean ± SD 6.19 ± 3.71), indicating a reduction in anxiety symptoms following resection (*p =* 0.024). In subgroup analyses (Table 2), nonanxious participants (HADS‐A < 8) did not show significant changes in HADS‐A scores postoperatively. However, anxious participants (HADS‐A ≥ 8) demonstrated a significant reduction in HADS‐A scores (*p =* 0.010). This subgroup effect largely accounts for the overall postoperative decrease in anxiety symptoms.

#### 3.3.3 HADS‐D

No significant differences were observed in HADS‐D scores for the entire cohort before (mean ± SD 4.57 ± 3.92) and after resection (mean ± SD 4.73 ± 3.51; *p =* 0.773). As shown in Table 2, in nondepressive participants (HADS‐D < 8), a trend toward higher postoperative scores was noted, suggesting a mild increase in depressive symptoms after resection, although this did not reach statistical significance. Conversely, participants with depressive symptoms (HADS‐D ≥ 8) showed a significant postoperative decrease in HADS‐D scores compared to preoperative levels (*p =* 0.012).

#### 3.3.4 Regression Analysis

The results were supported by regression analysis (Table 3), which examined the impact of preoperative HADS scores on postoperative change. All independent variables—except preoperative HADS‐D on the change in HADS‐A—showed a significant effect on the respective outcomes. This suggests that higher preoperative HADS scores (total, HADS‐A, and HADS‐D) predicted a greater reduction in symptoms 3 months after resection.

**TABLE 3:** Linear regression analysis for changes in HADS total score, HADS‐D, and HADS‐A.

	Regression coefficient *b*	*R* ^2^	95% CI	*T*	*p*
Change in HADS total score					
Preoperative HADS total score	−0.446	0.246	−0.715 to −0.178	−3.378	0.002**
Preoperative HADS‐D	−0.566	0.125	−1.080 to −0.053	−2.238	0.032*
Preoperative HADS‐A	−0.942	0.310	−1.423 to −0.460	−3.969	< 0.001***
Change in HADS‐D					
Preoperative HADS total score	−0.256	0.278	−0.398 to −0.114	−3.667	< 0.001***
Preoperative HADS‐D	−0.473	0.299	−0.721 to −0.224	−3.865	< 0.001***
Preoperative HADS‐A	−0.376	0.169	−0.661 to −0.090	−2.670	0.011*
Change in HADS‐A					
Preoperative HADS total score	−0.190	0.119	−0.368 to −0.013	−2.175	0.036*
Preoperative HADS‐D	−0.094	0.009	−0.428 to 0.241	−0.568	0.574
Preoperative HADS‐A	−0.566	0.299	−0.864 to −0.269	−3.864	< 0.001***

*Note*: Linear regression models with preoperative scores as predictors of postoperative change.

Abbreviations: b, unstandardized regression coefficient; CI, confidence interval; HADS, Hospital Anxiety and Depression Scale; HADS‐A, anxiety subscale; HADS‐D, depression subscale; *R* ^2^, coefficient of determination; *T*, *t*‐statistics.

*p ≤ 0.05.

**p ≤ 0.01.

***p ≤ 0.001.

### 3.4 Lateralization

When comparing tumor lateralization with affective symptoms, no significant differences were found. However, a trend was observed indicating that participants with tumors in the left hemisphere had lower preoperative HADS total and subscores than participants with tumors in the right hemisphere (*n* = 36, HADS total score ± SD: 9.42 ± 6.45 for the left hemisphere vs. 12.88 ± 6.95 for the right hemisphere, *p =* 0.130; HADS‐D ± SD: 4.11 ± 3.64 for the left hemisphere vs. 5.35 ± 4.19 for the right hemisphere, *p =* 0.345; HADS‐A ± SD: 5.32 ± 3.47 for the left hemisphere vs. 7.53 ± 3.50 for the right hemisphere, *p =* 0.065). Postoperatively, no significant differences were observed between right‐ and left‐hemispheric tumors for HADS total or subscores, although the same trend toward lower scores in left‐sided tumors persisted (Supporting Information 2). These results indicate that participants with a left‐hemispheric GBM tend to have fewer depressive and anxiety symptoms than participants with a right‐hemispheric GBM. Furthermore, within the left‐hemispheric group, a significant pre‐ to postoperative reduction in HADS‐A scores was observed, indicating a decrease in anxiety symptoms after resection (mean 5.32 vs. 3.74, *p =* 0.042) (Supporting Information 3).

### 3.5 Tumor Location

**FIGURE 2:**
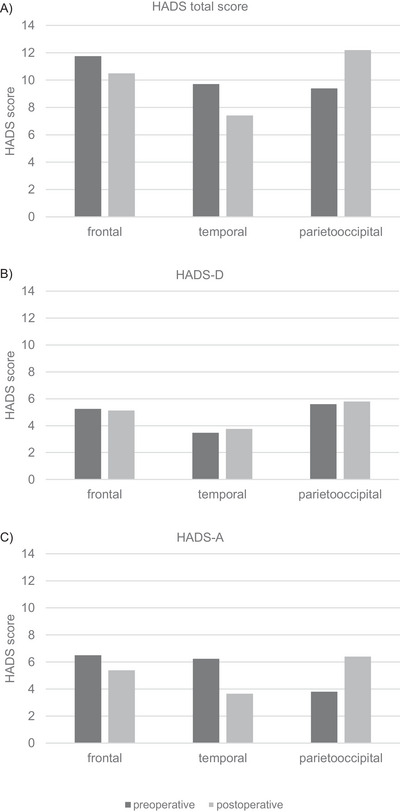
Pre‐ to postoperative changes in affective symptoms (HADS‐scores) by tumor location. (A) Total score, (B) depression subscale, and (C) anxiety subscale.

Figure 2 and Table 4 illustrate that participants with GBMs in the temporal lobe had significantly more anxiety symptoms right before resection compared to 3 months postoperatively (*p =* 0.009). In contrast, participants with tumors in the parieto‐occipital lobe demonstrated the opposite, showing a significant increase in affective symptoms postoperatively, as measured by the HADS total score (*p =* 0.045). This increase was mainly caused by the HADS‐A subscale (*p =* 0.057). No significant pre‐ to postoperative differences were observed in participants with tumors localized in the frontal lobe. Pairwise comparisons between tumor locations showed no significant differences in HADS scores at baseline or at 3‐month follow‐up (Supporting Information 4).

**TABLE 4:** Statistical analysis of HADS total score and subscores pre‐ and postoperatively based on tumor location (lobes).

	Frontal lobe (*n* = 8)				Temporal lobe (*n* = 17)				Parieto‐occipital lobe (*n* = 5)			
	Preoperative score	Postoperative score	Change in score (pre‐ vs. postoperative)		Preoperative score	Postoperative score	Change in score (pre‐ vs. postoperative)		Preoperative score	Postoperative score	Change in score (pre‐ vs. postoperative)	
	Mean ± SD	Mean ± SD	*T*	*p*	Mean ± SD	Mean ± SD	*T*	*p*	Mean ± SD	Mean ± SD	*T*	*p*
HADS total score	11.75 ± 9.63	10.50 ± 7.11	0.41	0.694	9.71 ± 6.58	7.41 ± 6.53	1.40	0.182	9.40 ± 5.51	12.20 ± 7.29	−2.89	0.045*
HADS‐D	5.25 ± 4.77	5.13 ± 3.27	0.083	0.936	3.47 ± 3.24	3.76 ± 3.21	−0.30	0.768	5.60 ± 4.56	5.80 ± 4.44	−0.54	0.621
HADS‐A	6.50 ± 5.18	5.38 ± 4.14	0.609	0.562	6.24 ± 3.75	3.65 ± 3.79	2.97	0.009**	3.80 ± 1.79	6.40 ± 3.21	−2.65	0.057

*Note*: Data are presented as mean ± SD. Paired *t*‐tests were used to compare pre‐ to postoperative scores within each tumor location group.

Abbreviations: HADS, Hospital Anxiety and Depression Scale; HADS‐D, depression subscale; HADS‐A, anxiety subscale; *T*, *t*‐statistic.

*p ≤ 0.05.

**p ≤ 0.01.

## 4 Discussion

In this study, we investigate participants with GBM regarding cognitive functions, affective symptoms, and tumor characteristics before and after resection. Our research is characterized by its exclusive focus on GBM. This allows a precise characterization of the cognitive and affective patterns specific to patients with this most aggressive brain tumor type, without confounding effects from heterogeneous glioma populations. Consequently, our findings can be interpreted with greater specificity for GBM patients. Our results suggest that cognitive performance in GBM patients remained without significant changes after resection, while affective symptoms changed depending on symptom severity and tumor characteristics.

### 4.1 Cognitive Performance

In our study, the mean MoCA total score was below the established cutoff of 24 points (Malek‐Ahmadi and Nikkhahmanesh 2024), indicating that most participants presented with mild cognitive impairment at baseline. Importantly, no specific MoCA subdomain was disproportionately affected, suggesting a global rather than domain‐specific cognitive deficit. Furthermore, the observed mean difference of 0.16 points between pre‐ and postoperative assessments falls well below the minimal clinically important difference of 2 points (Wong et al. 2017), reinforcing that the changes observed were not clinically meaningful.

The current evidence on cognition in GBM is limited. Only a few studies have specifically investigated GBM patients, and even fewer have assessed cognition both pre‐ and postoperatively within the same cohort using the MoCA as a screening tool. Most of the available data come from a more heterogeneous cohort of participants with high‐grade gliomas or brain tumors in general. Within this literature, the findings are controversial. Some studies report similarly low preoperative MoCA scores (Bondari et al. 2017), which is consistent with our results, while others describe slightly higher average scores close to or above the threshold, indicating less severe impairment (Jammula et al. 2022; Lorimer et al. 2019). In addition, postoperative outcomes vary considerably between studies: while several studies found stable cognitive function after resection (Dallabona et al. 2017; Habets et al. 2014), others showed a significant decline (Lang et al. 2017), selective improvements in certain areas (Ng et al. 2019), or mixed results in cognitive outcomes (Rijnen et al. 2020; Sinha et al. 2020; Kirkman et al. 2022). Overall, the current literature does not provide a consistent answer regarding the postoperative course of cognitive performance in GBM patients.

Several factors may explain discrepancies with previous studies, including tumor location, surgical approach, extent of resection, and patient‐related factors such as baseline performance, comorbidities, and age. In our cohort, cognitive performance remained stable 3 months after resection, which may reflect advances in neurosurgical techniques emphasizing functional preservation (Duffau 2011). Regarding the possible age effect, although the average age of our patients was not lower than in comparable studies, age‐related susceptibility to cognitive decline may still have contributed to the overall low baseline performance. Notably, age showed an influence on preoperative scores, but this effect was no longer evident for postoperative performance or changes over time, suggesting that age did not drive the observed stability in cognitive outcomes in our analysis.

The absence of significant, or clinically relevant, cognitive changes should also be interpreted with caution, given the relatively small sample size and the short follow‐up period. Three months may not be sufficient to detect delayed effects, especially since all participants received adjuvant chemoradiotherapy, which has been associated with subsequent cognitive decline (Crossen et al. 1994; Chapman et al. 2016; Correa et al. 2018; Wefel et al. 2004). Longer follow‐up studies, particularly in GBM cohorts with pre‐ and postoperative assessments, are therefore essential.

Beyond temporal limitations, it should also be noted that even within a homogeneous cohort with histopathologically confirmed GBM according to the WHO 2021 classification (diffuse glioma grade 4, IDH wild‐type), substantial interindividual variability in neurocognitive outcomes exists, driven by differences in various tumor characteristics. In particular, patients with tumors affecting language areas may experience distinct postoperative trajectories. In our cohort, a slight but nonsignificant decline in language scores was observed, which may reflect the susceptibility of language networks to surgical intervention. Importantly, the absence of clinically meaningful domain‐specific deterioration across all MoCA subdomains suggests that modern function‐preserving neurosurgical techniques may mitigate major postoperative cognitive losses.

### 4.2 Affective Symptoms

In our analysis, anxiety symptoms significantly decreased 3 months after resection, while depressive symptoms remained largely unchanged in the overall cohort. This suggests that GBM resection may alleviate anxiety‐related distress, possibly by reducing preoperative uncertainty and fear associated with the expected surgery. In addition, the psychological burden of such a diagnosis may decrease over time as patients develop adaptive coping strategies and acceptance. Previous studies in brain tumor patients have demonstrated that coping mechanisms such as problem‐oriented strategies, meaning‐making, and social support contribute significantly to reducing emotional distress and improving psychological adjustment (Guariglia et al. 2021; Baba and Adali 2021).

Only a few studies have reported a reduction in anxiety symptoms after brain tumor resection (Bunevicius et al. 2013; Rowe et al. 2025). To our knowledge, this is the first study to examine the course of affective symptoms before and after resection in a cohort consisting exclusively of GBM patients. This is particularly important because GBM differs from other brain tumors not only in terms of prognosis and aggressiveness but also in its rapid clinical course and high psychosocial burden, which cannot be extrapolated from mixed brain tumor cohorts.

Notably, participants with preoperative depressive or anxiety symptoms experienced a significant postoperative reduction in severity, whereas those without affective symptoms showed only minimal changes and tended to worsen within 3 months after resection. This pattern suggests that participants with more severe preoperative affective symptoms benefit most from resection, whereas those with few initial symptoms may experience worsening of their mood postoperatively.

Our regression analysis supports this interpretation, showing that higher preoperative HADS scores were associated with greater symptom reduction at follow‐up. Possible explanations include, as mentioned before, psychological relief after completing initial treatment, the gradual effect of coping strategies in highly distressed patients, and—conversely—a delayed adjustment phase in patients with low initial symptom awareness that could explain the results. Finally, the removal of tumor tissue may have a direct neurophysiological effect on mood regulation systems, if the affected tissue and resection site are functionally responsible for the processing and regulating of affect in the brain (Richter et al. 2015).

### 4.3 Tumor Lateralization

In our cohort, participants with GBMs in the right hemisphere tended to show more affective symptoms compared to those with left‐hemispheric tumors both pre‐ and postoperatively. This trend is consistent with previous studies suggesting that lesions in the right hemisphere may be more strongly associated with affective dysregulation (Álvarez‐Fernández et al. 2023). Mainio et al. also reported that primary brain tumors in the right hemisphere were associated with increased anxiety symptoms, which improved after resection (Mainio et al. 2003). This trend of postoperative improvement in anxiety symptoms was also observed in our study, although not statistically significant. Nevertheless, these findings support the hypothesis that the right hemisphere plays a dominant role in affective processing and emotional regulation (Sinha et al. 2020; Gainotti 2012). The trend observed in our study could therefore reflect disturbances in these regulatory networks, possibly also through direct neurophysiological effects on affective circuits. However, it should be noted that the observed differences did not reach statistical significance, most likely due to the small sample size.

### 4.4 Tumor Location

Depending on the location of the GBM, differences in pre‐ and postoperative anxiety levels were observed. Although pairwise comparisons between tumor locations showed no significant differences in affective symptoms at baseline or follow‐up, within‐group analyses revealed location‐specific trajectories. Participants with GBM in the temporal lobe exhibited higher preoperative anxiety symptoms compared to the follow‐up examination, suggesting that tumor‐related effects on temporal lobe structures may contribute to greater preoperative anxiety. The temporal lobe is part of the limbic system and is involved in affective processing, particularly in fear responses (Phelps and LeDoux 2005; Funayama et al. 2001). Progressive tumor growth in this region may impair the networks that support fear regulation, which manifests clinically as anxiety. Surgical resection, combined with the postoperative resolution of peritumoral edema, may alleviate the mass effect and restore the functional integrity of the neuroanatomical pathways involved in affective processing.

In contrast, participants with GBM in the parieto‐occipital region showed increased postoperative affective symptoms. Although these regions are not classically associated with emotion regulation, they are crucial for visuospatial processing, attention, and body awareness (Cavanna and Trimble 2006; Townsend and Courchesne 1994; Singh‐Curry and Husain 2009). A disruption of attentional networks, for example, due to mass effect, may indirectly lead to an exacerbation of anxiety, as impaired attention control has been shown to be associated with higher anxiety levels, especially under high cognitive demands (Shi et al. 2019). Neuroimaging studies further indicate that trait anxiety correlates with altered connectivity in the brain's attention networks (De la Peña‐Arteaga et al. 2024). Additionally, emotional distress can also arise from psychological reactions to functional deficits such as visual field cuts, which are common in parieto‐occipital lesions.

### 4.5 Clinical Implications and Future Directions

Our findings have several clinical implications for the management of GBM patients. The stability of cognitive performance 3 months after resection suggests that resection can be achieved without significant short‐term decline, supporting the principle of functional preservation alongside oncological goals (Duffau 2011). However, future studies including detailed surgical parameters are needed to clarify this balance.

With regard to affective symptoms, the observed bidirectional pattern—improvement in patients with elevated preoperative distress and worsening in those without symptoms—highlights the importance of systematic psychological assessment before and after resection. Routine screening can help identify patients at risk, with high‐burden patients benefiting from reassurance and low‐burden patients requiring close monitoring. While HADS is a useful screening tool, more specific instruments may allow for targeted interventions (Love et al. 2004; Vedana et al. 2002). Finally, the observed trend toward increased affective burden in patients with right‐hemispheric or parieto‐occipital tumors points to a potential role of these regions in emotional regulation. Patients with tumors in these areas may benefit from more intensive psychological monitoring and support to complement routine screening. Future research should further explore these neuroanatomical associations and clarify their clinical relevance, ideally in larger longitudinal GBM cohorts with longer follow‐up periods.

### 4.6 Limitations

Some limitations of our study should be acknowledged. The sample size (*n* = 37) was small, which limited the statistical power of the subgroup analyses and therefore reduced the ability to detect subtle effects. No formal a priori power analysis was conducted due to the exploratory nature of this study and the rarity of the condition under investigation. As a result, the study may have been underpowered to detect small but potentially meaningful effects, particularly in the subgroup analyses. The findings should therefore be interpreted as hypothesis‐generating and require confirmation in larger, adequately powered studies. Further, no correction for multiple comparisons was applied to the exploratory subgroup analyses, which increases the risk of type I errors. Future confirmatory studies with larger sample sizes should address this limitation.

Second, the follow‐up period was restricted to 3 months after resection, which does not allow conclusions about the long‐term trajectory of cognitive or affective outcomes. Moreover, the 3‐month assessment coincided with the period of adjuvant chemoradiotherapy, which may have independently influenced cognitive and affective outcomes. However, all participants received the same standardized adjuvant treatment (Stupp protocol), ensuring uniform treatment exposure across the cohort, and treatment‐related cognitive and affective effects are known to become more apparent months to years after treatment (Ng et al. 2019; Sinha et al. 2020; Kirkman et al. 2022; Duffau 2011). Notably, if adjuvant therapy had already exerted strong early detrimental effects, one would have expected cognitive decline at 3 months rather than the stability observed in our data. Future studies including multiple assessment time points (e.g., immediate postoperative, 3, 6, and 12 months) are needed to disentangle the respective contributions of resection and adjuvant treatment to the observed trajectories.

Third, affective symptoms were assessed by self‐report, which may have introduced response bias. Functional status was assessed with the KPS in our cohort, but no significant pre‐ to postoperative change was observed, limiting its value as an explanatory factor.

Additionally, molecular tumor features (e.g., MGMT promoter methylation status) and detailed surgical parameters (e.g., extent of resection, use of awake craniotomy) were not systematically included in the analysis. These factors could potentially contribute to the heterogeneity of cognitive and affective outcomes within the GBM population and should be considered in future studies. Furthermore, the MoCA, while well‐validated as a screening tool, may not detect subtle domain‐specific neuropsychological impairments. More detailed neuropsychological test batteries would be valuable in future studies to capture fine‐grained cognitive changes, particularly in language and executive functions.

Finally, social support has been shown to play a protective role against anxiety and depression in brain tumor patients (Cubis et al. 2019), but this factor was not included in our analyses. Future studies should therefore address these limitations by including larger sample sizes with extended follow‐up periods and additionally integrating the aforementioned factors to provide a more comprehensive understanding of affective symptom changes in GBM patients and potentially confirm the observed trends.

## 5 Conclusion

We demonstrated that GBM resection preserved but did not improve cognitive performance at 3 months of follow‐up. In contrast, the outcome of affective symptoms varied: participants with preoperative distress showed improvement, whereas those without symptoms tended to worsen.

Our analysis showed that tumor lateralization and location of the tumor modulated these trajectories, underscoring the importance of including tumor characteristics in psychological assessment.

A brief screening before and after resection can help identify patients at risk based on preoperative symptom burden. More specific tools are then needed to distinguish anxiety from depression and, therefore, enable targeted interventions. Integrating such monitoring into GBM treatment can improve patient support and quality of life.

## Author Contributions

**David Bellut**: writing – review and editing, resources, data curation, supervision, investigation, conceptualization, methodology, project administration, validation. **Carina E. Tschirky**: writing – original draft, writing – review and editing, resources, formal analysis, investigation, visualization, software, data curation, methodology. **Andre Richter**: supervision, data curation, conceptualization, funding acquisition, writing – review and editing, methodology, investigation, project administration, resources, validation. **Nicolin Hainc**: writing – review and editing, resources, data curation, investigation, methodology. **Anne‐Katrin Hickmann**: project administration, investigation, methodology, writing – review and editing, data curation, supervision, resources. **Ramona Hauser**: investigation, methodology, formal analysis, resources, data curation, writing – review and editing.

## Funding

This study was funded by the Müller‐Hartmann Stiftung Zürich, OPO‐Stiftung Zürich, Olga Mayenfisch Foundation, and a grant by the Swiss Cancer Research (KFS‐4270‐08‐2017).

## Ethics Statement

The study was approved by the Ethics Committee of Zurich (project number 2018‐00024).

## Consent

Informed consent was obtained from all participants before participation.

## Conflicts of Interest

The authors declare no conflict of interest.

## Supporting information




**Supplementary Material**: brb371478‐sup‐0001‐SuppMat.docx

**Supplementary Material**: brb371478‐sup‐0002‐SuppMat.docx

**Supplementary Material**: brb371478‐sup‐0003‐SuppMat.docx

**Supplementary Material**: brb371478‐sup‐0004‐SuppMat.docx

## Data Availability

The data that support the findings of this study are available from the corresponding author upon reasonable request.
