# Supplementary material for: Effects of Glioblastoma Resection on Cognitive Function and Affective Symptoms at Three‐Month Follow‐Up
Source: Brain Behav. 2026 May 5;16(5):e71478. doi: 10.1002/brb3.71478 (PMC13145357; doi:10.1002/brb3.71478)
Supplement: Supplementary file 1 — Supplementary Material: brb371478‐sup‐0001‐SuppMat.docx [file BRB3-16-e71478-s001.docx]

|  | Regression coefficient b | R^2^ | T | p |
| --- | --- | --- | --- | --- |
| Preoperative MoCA total score | -0.229 | 0.155 | -2.538 | 0.016* |
| Postoperative MoCA total score | -0.115 | 0.037 | -1.152 | 0.257 |
| Change in MoCA total score (pre- to postoperative) | -0.114 | 0.037 | 1.163 | 0.253 |

**Supplement 1: Linear Regression Analysis for the Effect of Age on the Pre- and Postoperative MoCA Score**

Linear regression models with age as predictor. b = unstandardized regression coefficient; R² = coefficient of determination; T = t-statistic; MoCA = Montreal Cognitive Assessment; * p ≤ 0.05
