# Supplementary material for: Effects of Glioblastoma Resection on Cognitive Function and Affective Symptoms at Three‐Month Follow‐Up
Source: Brain Behav. 2026 May 5;16(5):e71478. doi: 10.1002/brb3.71478 (PMC13145357; doi:10.1002/brb3.71478)
Supplement: Supplementary file 2 — Supplementary Material: brb371478‐sup‐0002‐SuppMat.docx [file BRB3-16-e71478-s004.docx]

|  | Hemisphere | |  |
| --- | --- | --- | --- |
|  | left (n = 19) | right (n = 17) |  |
|  | Mean ± SD | Mean ± SD | p |
| Postoperative HADS total score | 8.00 ± 5.48 | 11.47 ± 7.49 | 0.119 |
| Postoperative HADS-D | 4.26 ± 3.03 | 5.47 ± 3.96 | 0.308 |
| Postoperative HADS-A | 3.74 ± 2.68 | 6.00 ± 4.15 | 0.058 |

**Supplement 2: Statistical Analysis of the Postoperative HADS Total Score and Sub Scores and the Lateralization of the Tumor**

Data are presented as mean ± SD. Independent t-tests were used to compare postoperative scores between left and right hemisphere tumor locations. HADS = Hospital Anxiety and Depression Scale; HADS-D = Depression subscale; HADS-A = Anxiety subscale.
