# Supplementary material for: Effects of Glioblastoma Resection on Cognitive Function and Affective Symptoms at Three‐Month Follow‐Up
Source: Brain Behav. 2026 May 5;16(5):e71478. doi: 10.1002/brb3.71478 (PMC13145357; doi:10.1002/brb3.71478)
Supplement: Supplementary file 3 — Supplementary Material: brb371478‐sup‐0003‐SuppMat.docx [file BRB3-16-e71478-s003.docx]

|  | Left Hemisphere (n = 19) | | | | | Right Hemisphere (n = 17) | | | | | | |
| --- | --- | --- | --- | --- | --- | --- | --- | --- | --- | --- | --- | --- |
|  | Preoperative score | Postoperative score | Change in score (pre- vs. postoperative) | | | | Preoperative score | Postoperative score | Change in score (pre- vs. postoperative) | | |  |
|  | Mean ± SD | Mean ± SD | T | 95%-CI | p (two-sided) | | Mean ± SD | Mean ± SD | T | 95%-CI | p (two-sided) |  |
| HADS total score | 9.42 ± 6.45 | 8.00 ± 5.48 | 1.194 | -1.08 – 3.92 | 0.248 | | 12.88 ± 6.95 | 11.47 ± 7.49 | 0.768 | -2.49 – 5.31 | 0.454 |  |
| HADS-D | 4.11 ± 3.64 | 4.26 ± 3.03 | -0.258 | -1.45 – 1.13 | 0.800 | | 5.35 ± 4.18 | 5.47 ± 3.96 | -0.115 | -2.28 – 2.05 | 0.910 |  |
| HADS-A | 5.32 ± 3.47 | 3.74 ± 2.68 | 2.185 | 0.061 – 3.10 | 0.042* | | 7.53 ± 3.50 | 6.00 ± 4.15 | 1.356 | -0.86 – 3.92 | 0.194 |  |

**Supplement 3: Pre- and Postoperative HADS Total- and Subscores in GBM Patients by Tumor Lateralization (Left vs. Right Hemisphere)**

Data are presented as mean ± SD. Paired t-tests were used to compare pre- and postoperative scores within each hemisphere group. HADS = Hospital Anxiety and Depression Scale; HADS-D = Depression subscale; HADS-A = Anxiety subscale; CI = Confidence Interval; T = t-statistic; * p ≤ 0.05.
