# Supplementary material for: Effects of Glioblastoma Resection on Cognitive Function and Affective Symptoms at Three‐Month Follow‐Up
Source: Brain Behav. 2026 May 5;16(5):e71478. doi: 10.1002/brb3.71478 (PMC13145357; doi:10.1002/brb3.71478)
Supplement: Supplementary file 4 — Supplementary Material: brb371478‐sup‐0004‐SuppMat.docx [file BRB3-16-e71478-s002.docx]

**Supplement 4: Pairwise Comparison of Affective Symptoms (HADS) by Tumor Location**

Preoperative Assessment

|  | Frontal vs. Temporal | Frontal vs. Parieto-Occipital | Temporal vs. Parieto-Occipital |
| --- | --- | --- | --- |
| HADS total score |  |  |  |
| Frontal, mean ± SD | 11.75 ± 9.63 | 11.75 ± 9.63 | — |
| Temporal, mean ± SD | 9.71 ± 6.58 | — | 9.71 ± 6.58 |
| Parieto-Occipital, mean ± SD | — | 9.40 ± 5.51 | 9.40 ± 5.51 |
| p-value | 0.539 | 0.632 | 0.926 |
| HADS-A |  |  |  |
| Frontal, mean ± SD | 6.50 ± 5.18 | 6.50 ± 5.18 | — |
| Temporal, mean ± SD | 6.24 ± 3.75 | — | 6.24 ± 3.75 |
| Parieto-Occipital, mean ± SD | — | 3.80 ± 1.79 | 3.80 ± 1.79 |
| p-value | 0.885 | 0.291 | 0.180 |
| HADS-D |  |  |  |
| Frontal, mean ± SD | 5.25 ± 4.77 | 5.25 ± 4.77 | — |
| Temporal, mean ± SD | 3.47 ± 3.24 | — | 3.47 ± 3.24 |
| Parieto-Occipital, mean ± SD | — | 5.60 ± 4.56 | 5.60 ± 4.56 |
| p-value | 0.283 | 0.898 | 0.252 |

Postoperative Assessment

|  | Frontal vs. Temporal | Frontal vs. Parieto-Occipital | Temporal vs. Parieto-Occipital |
| --- | --- | --- | --- |
| HADS total score |  |  |  |
| Frontal, mean ± SD | 10.50 ± 7.11 | 10.50 ± 7.11 | — |
| Temporal, mean ± SD | 7.41 ± 6.53 | — | 7.41 ± 6.53 |
| Parieto-Occipital, mean ± SD | — | 12.20 ± 7.29 | 12.20 ± 7.29 |
| p-value | 0.294 | 0.686 | 0.175 |
| HADS-A |  |  |  |
| Frontal, mean ± SD | 5.38 ± 4.14 | 5.38 ± 4.14 | — |
| Temporal, mean ± SD | 3.65 ± 3.79 | — | 3.65 ± 3.79 |
| Parieto-Occipital, mean ± SD | — | 6.40 ± 3.21 | 6.40 ± 3.21 |
| p-value | 0.312 | 0.648 | 0.157 |
| HADS-D |  |  |  |
| Frontal, mean ± SD | 5.13 ± 3.27 | 5.13 ± 3.27 | — |
| Temporal, mean ± SD | 3.76 ± 3.21 | — | 3.76 ± 3.21 |
| Parieto-Occipital, mean ± SD | — | 5.80 ± 4.44 | 5.80 ± 4.44 |
| p-value | 0.336 | 0.757 | 0.265 |

Data are presented as mean ± SD. Independent t-tests were used for pairwise comparisons between tumor locations both pre- and postoperative. HADS = Hospital Anxiety and Depression Scale; HADS-D = Depression subscale; HADS-A = Anxiety subscale.
